# Relative Effectiveness of Social Media, Dating Apps, and Information Search Sites in Promoting HIV Self-testing: Observational Cohort Study

**DOI:** 10.2196/35648

**Published:** 2022-09-23

**Authors:** Chrysovalantis Stafylis, Gabriella Vavala, Qiao Wang, Bethany McLeman, Shea M Lemley, Sean D Young, Haiyi Xie, Abigail G Matthews, Neal Oden, Leslie Revoredo, Dikla Shmueli-Blumberg, Emily G Hichborn, Erin McKelle, Landhing M Moran, Petra Jacobs, Lisa A Marsch, Jeffrey D Klausner

**Affiliations:** 1 Department of Population and Public Health Sciences University of Southern California Los Angeles, CA United States; 2 Division of Infectious Diseases, David Geffen School of Medicine University of California Los Angeles Los Angeles, CA United States; 3 Geisel School of Medicine Dartmouth College Lebanon, NH United States; 4 Department of Emergency Medicine, School of Medicine University of California, Irvine Irvine, CA United States; 5 Department of Informatics, Bren School of Information and Computer Sciences University of California, Irvine Irvine, CA United States; 6 The Emmes Company LLC Rockville, MD United States; 7 Education, Training and Research Associates Oakland, CA United States; 8 National Institute on Aging Bethesda, MD United States

**Keywords:** HIV prevention, PrEP, home HIV test, social media, dating apps, search engines, HIV, human immunodeficiency virus, self-testing, infection, digital health, health promotion, MSM, pre-exposure prophylaxis, medical information

## Abstract

**Background:**

Social media sites, dating apps, and information search sites have been used to reach individuals at high risk for HIV infection. However, it is not clear which platform is the most efficient in promoting home HIV self-testing, given that the users of various platforms may have different characteristics that impact their readiness for HIV testing.

**Objective:**

This study aimed to compare the relative effectiveness of social media sites, dating apps, and information search sites in promoting HIV self-testing among minority men who have sex with men (MSM) at an increased risk of HIV infection. Test kit order rates were used as a proxy to evaluate promotion effectiveness. In addition, we assessed differences in characteristics between participants who ordered and did not order an HIV test kit.

**Methods:**

Culturally appropriate advertisements were placed on popular sites of three different platforms: social media sites (Facebook, Instagram), dating apps (Grindr, Jack’D), and information search sites (Google, Bing). Advertisements targeted young (18-30 years old) and minority (Black or Latinx) MSM at risk of HIV exposure. Recruitment occurred in 2 waves, with each wave running advertisements on 1 platform of each type over the same period. Participants completed a baseline survey assessing sexual or injection use behavior, substance use including alcohol, psychological readiness to test, attitudes toward HIV testing and treatment, and HIV-related stigma. Participants received an electronic code to order a free home-based HIV self-test kit. Follow-up assessments were conducted to assess HIV self-test kit use and uptake of pre-exposure prophylaxis (PrEP) at 14 and 60 days post enrollment.

**Results:**

In total, 271 participants were enrolled, and 254 were included in the final analysis. Among these 254 participants, 177 (69.7%) ordered a home HIV self-test kit. Most of the self-test kits were ordered by participants enrolled from dating apps. Due to waves with low enrollment, between wave statistical comparisons were not feasible. Within wave comparison revealed that Jack’D showed higher order rates (3.29 kits/day) compared to Instagram (0.34 kits/day) and Bing (0 kits/day). There were no associations among self-test kit ordering and HIV-related stigma, perceptions about HIV testing and treatment, and mistrust of medical organizations.

**Conclusions:**

Our findings show that using popular dating apps might be an efficient way to promote HIV self-testing. Stigma, perceptions about HIV testing and treatment, or mistrust of medical organizations may not affect order rates of HIV test kits promoted on the internet.

**Trial Registration:**

ClinicalTrials.gov NCT04155502; https://clinicaltrials.gov/ct2/show/NCT04155502

**International Registered Report Identifier (IRRID):**

RR2-10.2196/20417

## Introduction

The incidence of HIV infection remains high among minority men who have sex with men (MSM) [1]. Frequent testing for HIV infection can identify new infections early, and it is essential in ending the HIV epidemic [2]. HIV self-testing is an alternative HIV screening method that is commercially available, approved by the Food and Drug Administration, and can reach individuals who have never tested before. It can reach populations at risk, such as Black and Latinx individuals, identify new cases of HIV infection [3-6], and lead individuals to seek additional HIV prevention options, such as testing for sexually transmitted infections or pre-exposure prophylaxis (PrEP) [7]. Prevention studies and public health programs have been adopting HIV self-tests [8,9] and combining them with new technologies, such as smartphone apps [10] or smart devices [4], to reach populations with high incidence of HIV infection, such as Black and Latinx MSM. Despite multiple efforts, the uptake of HIV testing remains inadequate, especially among individuals at high risk for HIV infection [11]. Thus, optimizing the promotion of HIV testing is important.

Due to their extensive popularity, social media sites and dating apps have been used to promote and recruit participants for HIV prevention research studies with high rates of success [5,12-14]. According to a recent Centers for Disease Control and Prevention (CDC) report reviewing HIV self-testing programs, 27 health departments and community organizations [9] used multiple platforms for promotion, mainly social media (19/27) followed by “traditional” printed media (9/27) and dating apps (6/27). Compared to in-person recruitment, web-based platforms have the capacity to reach a high number of difficult-to-reach populations and individuals at risk [5,14,15], overcoming stigma or other logistic obstacles [15,16] in a cost-efficient manner [16,17]. Indeed, the New York Department of Public Health used advertisements on social media, dating apps, and websites to reach 28,921 users, identifying 17,383 eligible MSM, transgender, and gender nonconforming individuals during its HIV self-testing campaign. Most of the participants were under the age of 35 years and identified as Black or Latinx. In addition, the first wave of this campaign reached 3359 users in only 23 days, distributing 2497 home test kit voucher codes to eligible users [18]. Social media and dating apps have been widely adopted as means of promoting HIV home testing. Although different from dating apps and social media sites, information search sites (eg, Google) are commonly used for seeking information on HIV testing and PrEP [19,20] and could represent a promising outreach avenue. Their use for enrollment and HIV testing promotion has not been evaluated.

However, little is known about the relative effectiveness of these different web-based platforms (ie, social media, dating apps, and information search sites) in promoting HIV self-testing. Parker et al [21] conducted a secondary analysis in a study enrolling substance-using sexual and gender minority adolescents and young adults to evaluate the efficacy of their enrollment strategy. The study used multiple methods to enroll participants, including social media platforms (Facebook, Instagram, Twitter, Tumblr), dating apps (Grindr, Scruff, Jack’D), internet-based health boards, and venue-based enrollment. They recorded 17,328 visits to the eligibility screener on the landing page, with a 36.2% (6274/17,328) screener survey completion ratio. Researchers identified 580 participants among those who consented and were eligible to participate (580/623, 93.1%), indicating a high recruitment proportion. The majority of their participants were enrolled from Facebook, Instagram, and Grindr. Studies and programs use these platforms based on the experience of previous studies and expert recommendations [22].

Data on the effectiveness of public health promotion through different platforms leading to testing or PrEP are missing. We can only infer the effectiveness of promotion indirectly, as head-to-head comparisons of the effectiveness of the different platforms and sites to reach individuals for public health promotion are missing. This would allow researchers and prevention programs to optimize their budget and strategy. The primary objective of this study was to compare ordering of HIV self-testing kits among users recruited through 3 different types of web-based platforms, including social media, dating apps, and information search sites. Test kit ordering was used as a proxy for analyzing the effectiveness of promoting HIV self-testing on different sites. The secondary goal was to evaluate the association of key moderating variables—substance use, psychological readiness to test, and perceptions and attitudes related to HIV testing—with the ordering of HIV self-testing kits.

## Methods

### Recruitment

In this longitudinal observational cohort study, advertisements promoting free HIV self-testing were placed on three platform types: social media (Facebook, Instagram), dating apps (Grindr, Hornet), and information search sites (Google, Bing) (Table S1 in Multimedia Appendix 1). The advertisements were organized in 2 “waves,” with each wave consisting of 1 social media website, 1 dating app, and 1 information search site. The Wave 1 (Facebook, Grindr, Google) recruitment stopped early, as Grindr unexpectedly stopped running all self-service platform advertisements (including the study advertisement) due to a change in corporate ownership [23,24]. We continued with Wave 2 (Instagram, Jack’D, Bing) as planned and a relaunched Wave 1 (Facebook, Grindr, Google) once Grindr access was restored.

Before launching each wave, we allocated the same amount of funds for each of the 3 sites and optimized them to run for at least 30 calendar days by dividing the available funds in the prespecified promotional period. However, due to slow enrollment during the COVID-19 pandemic, we extended the second phase of Wave 1 up to 63 days. The advertisement used on social media and dating apps was an image that included a person and text (“Get a FREE HIV test”), whereas promotional keywords related to HIV testing and PrEP were selected for information search sites (as images are not allowed). The same image and keywords were used in all waves. The advertisements were launched in the District of Columbia (DC) and 8 states (Florida, Georgia, Louisiana, Maryland, Mississippi, Nevada, South Carolina, and Texas), which were selected based on their high incidence of HIV infection. More information regarding the promotional campaign can be found in the published protocol [25].

Upon clicking on the study advertisement, website users landed on the study information page, where they received general information about the study, underwent eligibility screening, and reviewed study procedures. Following electronic informed consent, participants completed the baseline assessment and were emailed a unique electronic code to order their HIV home self-test kit through Orasure.com (Bethlehem, PA). Participants also received an electronic coupon for a free telemedicine PrEP visit. Participants were followed up at 14 and 60 days after enrollment. At follow-up, participants were asked about their HIV self-test use and self-test results; depending on their self-test result, they were asked if they visited a PrEP provider and started PrEP, as well as their opinions on PrEP. If they tested positive for HIV antibodies with the home self-test kit, they were asked if they had visited a clinic for confirmatory testing and HIV treatment. In addition, we tracked test kit orders through automated reports, collected anonymous advertisement metrics through the web applications of the platforms, and recorded the costs for each promotion site and wave.

### Inclusion Criteria

We enrolled MSM aged 18-30 years who identified as Latinx or Black/African American people (including multiracial and multiethnic individuals of these groups); they reported having condomless anal sex in the past 90 days or having more than 1 male sex partner in the past 90 days. Participants were considered ineligible if they were HIV-positive, if they were tested for HIV infection in the past 90 days, and if they were taking PrEP currently or at any time during the past 6 months before enrollment.

### Outcome

The primary outcome was the number of HIV self-test kits ordered per day through each type of internet-based platform (social media, dating, information site) during the period in which each wave was operational. As a secondary outcome, we explored the association of reported substance use, stage of change for HIV testing based on the transtheoretical model, attitudes toward HIV testing and treatment, HIV-related stigma, medical mistrust, and opinions about PrEP measures and self-test kit ordering. As an exploratory outcome, we recorded the advertisement metrics of each campaign to measure differences in the reach and cost.

### Assessments

Study assessments are described in the protocol [25]. Participants were asked to self-report test kit and PrEP use. We calculated the substance-specific TAPS (Tobacco, Alcohol, Prescription medication, and other Substance) tool score [26] of each participant. For each substance, a score of 1 was classified as “problem use” (low-risk substance use), whereas a score of 2 or higher was classified as “high-risk substance use.” We collected participants’ opinions about HIV treatment using a 10-item questionnaire [27]. Each question was presented as a visual analog scale (eg, slider) with “strongly disagree” and “strongly agree” anchoring the 2 extremes. We assumed an underlying continuous, linear relationship between the 2 anchors, and data for opinions about HIV treatment are presented as the mean score for each question with its SD. PrEP opinions, barriers, and facilitators were collected using a 5-point Likert scale (ranging from “Not at all important” to “Extremely important”).

Finally, we monitored the performance of the promotional campaigns using the impressions (number of times the advertisement is shown on a screen), clicks (number of times the advertisement was “clicked”), click-through rate (clicks/impressions), and funds spent.

### Statistical Analysis

Participants who were enrolled from Google and Facebook while Grindr was inactive (early during Wave 1) were excluded from analyses. This ensured that we included data when all 3 sites were active and thus had an equal chance to enroll participants. Participants who did not order a test kit within 60 days of the test code being emailed to them were classified as “not ordered a self-test kit.” The 2 advertisement periods of Wave 1 were combined before analysis. Prior to statistical modeling, the number of HIV home self-test kits ordered from each platform, specific platform types (sites), and number days of recruitment in each wave were summarized. In addition, the observed daily self-test kit order rates for each site and platform type were calculated (order rate = number of orders / number of advertising days during each wave).

Per our primary research question, we intended to determine the statistical difference in the self-test kit ordering rates by platform type (social media, information search site, and dating app) using a Poisson regression model; however, due to significant platform-by-wave interactions and widely differing order rates between sites within the same platform, it was not appropriate to combine or pool sites across the same platform for statistical evaluation of the platform difference. Therefore, we compared the specific platform differences in terms of the order rates within the same wave. We conducted pairwise comparison for all 6 sites from the 2 waves with multiple testing adjustments using the Hochberg method [28].

Demographic and baseline characteristics were presented using summary statistics. Continuous variables were summarized using percentiles (median, and 25th and 75th percentiles), and means with their SDs. Categorical variables were summarized with frequencies and percentages. To assess differences in the measures between participants who ordered a test kit and those who did not order a test kit, we used the Student *t* test for continuous variables, Fisher exact test for categorical variables, and Wilcoxon rank sum test for Likert responses. Data analysis was carried out using Statistical Analysis Software (version 9.4, SAS Institute).

### Adaptations Due to COVID-19 Pandemic

We conducted a third wave of promotion and enrollment on Twitter, Yahoo, and Hornet. This wave was conducted between April 6, 2020, and May 6, 2020, during the first days of the public health emergency proclamation. Despite the promotional waves being active, no participants were enrolled, and no test kits were ordered during Wave 3, which made our statistical model inestimable. As enrollment during this period does not reflect “expected conditions” and scientific comparisons would not be accurate, we decided to exclude Wave 3 from all the analyses.

### Sensitivity Analyses

We conducted 3 sensitivity analyses using the statistical approach, Poisson regression, and posthoc contrast. The primary sensitivity analysis included any self-test kits ordered at any time during the study (ie, outside of the 60-day window for the primary analysis) and by any participants in the validated participant population. The second sensitivity analysis attempted to address the fact that Wave 1 occurred in 2 phases because 1 promotional platform (Grindr) stopped all advertising. The final sensitivity analysis assessed the impact of the COVID-19 pandemic.

### Missing Data

The analysis of primary outcomes does not include missing data. In questions where participants could “skip” and not respond, the answer was classified as “missing” and was not included in the calculation of those variable frequencies.

### Ethics Approval

This study (trial registration: NCT04155502) was reviewed and approved by the Institutional Review Board at the University of California, Los Angeles (IRB #18-001580).

## Results

### Baseline Characteristics

Between January and September 2020, a total of 10,669 individuals visited the study website, directed from study advertisements placed on the platform sites across all waves. During the study period, 254 participants were enrolled from 6 platform sites. The majority were enrolled from urban areas of Texas, Florida, DC, and Georgia. The average age (SD) of participants was 24.4 years (SD 3.7 years). Most (199/254, 78.4%) participants identified as Black/African American and 26% (66/254) reported that they were Latinx.

The median number of sex partners in the past 90 days was 4 (IQR 3-6). Among the 254 participants, 210 (82.7%) participants reported receptive condomless anal sex during the past 90 days. Only 23 (8.9%) participants received PrEP before. When asked about condom use, 5 (2%) reported that they always used a condom, whereas 36 (14.2%) said that they never used condoms. Most of the participants (191, 75.2%) tested for HIV infection in the past. Among those tested in the past, the median (IQR) time since their last test was 11 months (6-21). Participants who never previously tested reported that their main reasons for not testing were their fear of obtaining a positive HIV result and their belief that HIV exposure was unlikely. Table 1 presents the participant demographics and behaviors.

**Figure 1:**
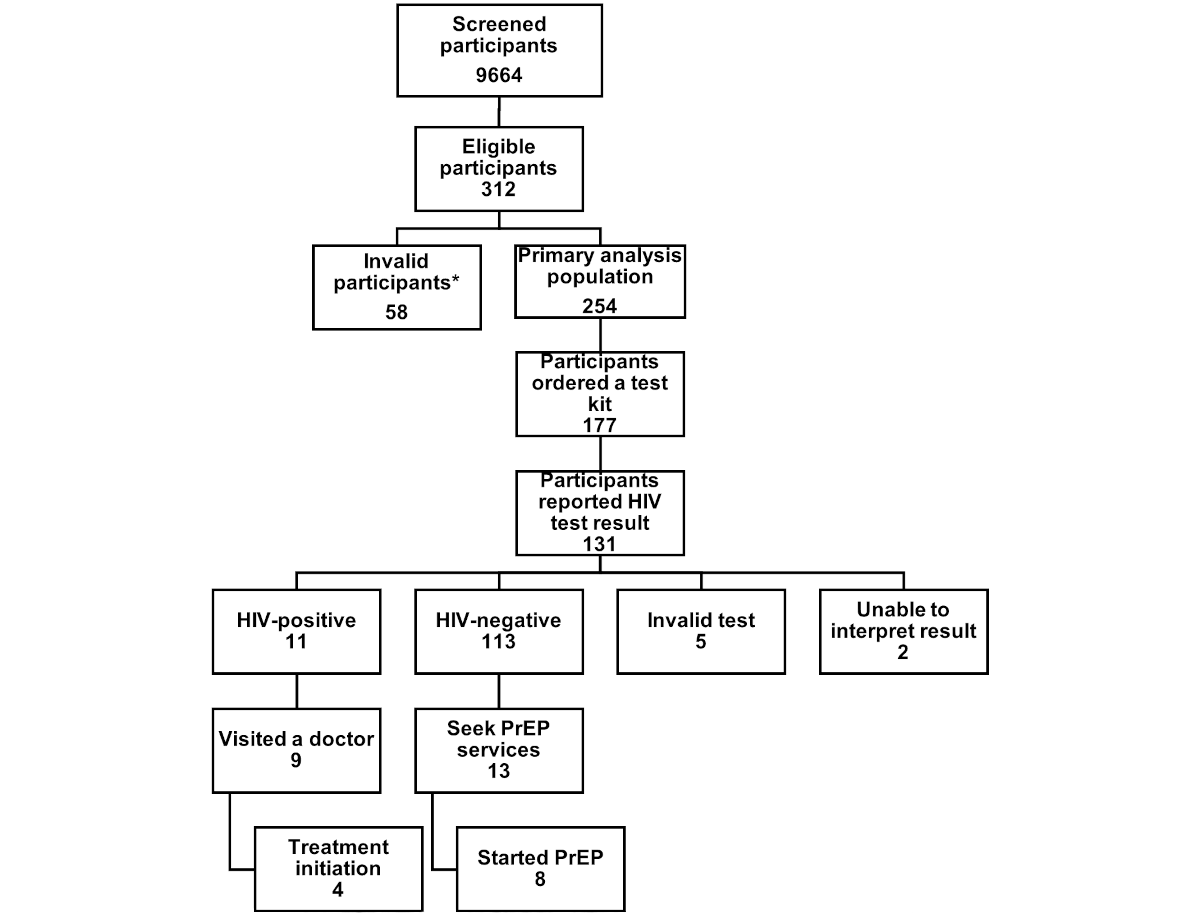
Enrollment, HIV home test kit use, pre-exposure prophylaxis uptake, and linkage to care among participants of the National Institute on Drug Abuse Clinical Trials Network Social Media PrEP Study, 2020. PrEP: pre-exposure prophylaxis. *Invalid participants include duplicate entries, fake accounts, and participants outside the country.

In terms of HIV home test kit use, 131 out of the 177 participants (74%) reported a self-test result, with 11 of the 131 participants (8.4%) reporting a positive HIV test result; 9 of these 11 (82%) reported that they sought confirmatory testing and 4 of these 9 (44.4%) had started treatment for HIV. Among the 120 participants who reported a negative test result for HIV infection, 13 (11%) reported visiting a provider to discuss PrEP or reported starting PrEP (Figure 1).

**Table 1:** Summary of National Institute on Drug Abuse Clinical Trials Network Social Media Pre-exposure Prophylaxis Study, 2020, population sociodemographic and behavioral characteristics (N=254).

Characteristic		Value
Age in years, median (IQR)		25 (23-27)
**Ethnicity, n (%)**		
	Hispanic/Latinx	66 (26)
**Race, n (%)**		
	American Indian or Alaskan Native	1 (0.4)
	Black or African American	196 (78.4)
	White	28 (11.2)
	Other	14 (5.6)
	Multiracial	11 (4.4)
**History of PrEP^a^ uptake, n (%)**		
	Never taken PrEP	232 (91.3)
	In the past 6 months	22 (8.9)
Number of male sex partners in the past 90 days, median (IQR)		4 (3-6)
**Condom use, n (%)**		
	Never	36 (14.2)
	Sometimes	108 (42.5)
	About half the time	37 (14.5)
	Most of the time	68 (26.8)
	Always	5 (2)
Condomless receptive anal sex in the past 90 days, n (%)		210 (82.7)
Ever tested for HIV during lifetime, n (%)		191 (75.2)
**If tested for HIV, median (IQR)**		
	Months since last HIV test	11 (6-21)
If not tested for HIV, n (%)		63 (24.8%)
**Main reasons cited by the 63 participants for not getting tested, n (%)**		
	Unlikely to be exposed to HIV	8 (12.7)
	Afraid of testing HIV-positive	26 (41.3)
	Did not want to think about HIV/HIV-positive	8 (12.7)
	Worried about names being reported if positive	3 (4.8)
	Dislike for needles	5 (8)
	Unable to trust that the results will be confidential	3 (4.8)
	Unaware of where to get tested	7 (11.1)
	Other reasons	3 (4.8)

^a^PrEP: pre-exposure prophylaxis.

### Primary Outcome

Table 2 summarizes the analysis results for the primary outcome. In total, 177 of the 254 participants ordered test kits during the study period. Overall, those recruited through dating apps had the highest order rate (1.24 kits/day), followed by social media platforms (0.24 kits/day) and information search platforms (0.16 kits/day; Table 2). Pairwise contrasts between the platforms showed that in Wave 1, there was no statistically significant difference across the specific platforms. Specifically, the Hockberg-adjusted *P*=.59 for all the following pairwise contrasts in Wave 1: Facebook (social media) vs Google (information site), Facebook (social media) vs Grinder (dating app), and Google vs Grinder contrasts (Note: False discovery–adjusted *P* values were different between each pair contrast but not significant.). However, in Wave 2, there was a statistically significant difference across the platforms with Jack’D (dating app) being the most effective site (3.29 kits/day), compared to Instagram (0.34 kits/day) and Bing (0 kits/day). Specifically, the Hockberg-adjusted *P*=.002 for Bing (information site) vs Instagram (social media) contrast; *P*<.001 for the Bing (Information site) vs Jack’D (dating app) contrast and for the Instagram vs Jack’D contrast. All 3 types of primary outcome sensitivity analyses showed results similar to the primary analysis (see Multimedia Appendix 2 for details).

**Table 2:** Number and rate of HIV home self-test kits ordered through promotional platforms by wave per protocol sample in the National Institute on Drug Abuse Clinical Trials Network Social Media Pre-exposure Prophylaxis Study, 2020 (N=254).

Type of platform		Wave	Number of days for each wave	Number of test kits ordered	Order rate (ordered test kits/day)
**Social media site**					
	Facebook	1^a^	70	13	0.19
	Instagram	2	38	13	0.34
	Subtotal	N/A	108	26	0.24
**Dating app**					
	Grindr	1^a^	70	9	0.13
	Jack'D	2	38	125	3.29
	Subtotal	N/A	108	134	1.24
**Information search site**					
	Google	1^a^	70	17	0.24
	Bing	2	38	0	0.00
	Subtotal	N/A	108	17	0.16
Total		N/A	108	177	1.64

^a^Wave 1: includes original Wave 1 data from the time when Google, Facebook, and Grindr were advertising simultaneously and the data from the second phase of Wave 1.

N/A: not applicable.

### Secondary Outcomes

We explored the association of HIV test kit ordering and factors that could potentially affect ordering a test kit (see Multimedia Appendix 3). We found no statistically significant associations between test kit ordering and substance use, stage of health behavior change regarding HIV testing, and medical mistrust. However, ordering a HIV test kit was associated with the statement “People in my life would leave if I had HIV;” 48.1% (37/77) did not order a test kit whereas 33.7% (59/175) ordered a test kit; *P*=.04). Participants who did not order a kit were more likely to agree with the statement “I think that new HIV/AIDS treatments can eradicate the virus from your body,” compared to those who ordered a kit (*P*=.03; Table (d) in Multimedia Appendix 3). People who ordered a self-test kit were more likely to disagree with the statement “I could not be friends with someone who has HIV/AIDS,” compared to those who did not order a kit (*P*=.03).

Of the 254 participants, 119 (46.8%) were classified as “high-risk alcohol use’” and 67 (26.4%) as “problem alcohol use.” Approximately 94 (37%) participants were classified as “high-risk cannabis use” and 19% of the participants as “problem cannabis use.” Over half (136=53.5%) of the study participants reported that they were ready to start regularly testing for HIV (“Determination” stage of change), but only a small proportion of participants (12/177, 6.8%) among those who ordered a kit and 7.8% (6/77) of those who did not order a kit reported testing regularly (“Maintenance” stage of change). In total, 60 of the 254 (23.6%) participants agreed with the statement ”I feel afraid of people living with HIV/AIDS” and only 9 (3.6%) agreed with the statement “I could not be friends with someone who has HIV/AIDS.” Many participants believed that mistakes are common in health care settings (155, 61.2%) and that organizations cover up their mistakes (153, 60.3%). They also reported being cautious toward health care organizations (151, 59.6%), with 159 (62.6%) feeling that patients have occasionally been misled or deceived by medical professionals.

Few participants (50/168, 29.8%) had a negative attitude toward taking PrEP (“I feel uncomfortable taking HIV medication when I don’t have HIV.”); some of them (21/165, 12.7%) were generally not ashamed to tell people (“I am ashamed to tell others that I am on PrEP.”). However, they expressed concerns over the cost and long-term health effects. The reported barriers to starting PrEP included potential adverse effects of the medication (117/164, 71.3%) and fear of HIV treatment failure because of PrEP in case they get infected with HIV (138/163, 54.3%; Figure S2 in Multimedia Appendix 3). Facilitators to starting PrEP included the following: getting free HIV and sexually transmitted infection testing (134/162, 82.3%), acquiring free or low-cost PrEP (127/164, 77.4%), receiving a recommendation for PrEP from their doctor (119/163, 73%), and receiving additional counseling and support while on PrEP (118/163, 72.4%) (Figure S3 in Multimedia Appendix 3).

### Performance of Advertisement Campaigns

Throughout the duration of the promotional campaign, we spent approximately US $20,000 in total per platform. Dating apps had the highest engagement (click-through rate of 4% resulting in 202 enrolled participants), even though they had the lowest number of impressions. Advertising through social media resulted in a high number of clicks (impressions) and low engagement (click-through rate of 0.6%). Information search sites recorded the lowest number of impressions among the 3 platform types and the lowest number of users who were enrolled in the study (n=19), as shown in Table 3. We calculated the cost per enrolled participant as US $491.6 for social media, US $88.8 for dating apps, and US $841 for information search sites.

**Table 3:** Performance of advertisements by platform throughout the advertisement campaign in the National Institute on Drug Abuse Clinical Trials Network Social Media PrEP Study, 2020.

Platform	Impressions^a^	Clicks^b^	Click-through rate (%)^c^	Users screened^d^	Enrolled participants	Total funds spent (US $)
Social media	3,864,778	21,399	0.6	2679	33	16,221.52
Dating apps	1,331,200	53,067	4	4390	202	17,939.40
Information search sites	708,770	10,869	1.5	2562	19	15,978.86

^a^Impressions refer to the number of times the advertisement is shown on a screen. A user may see the same advertisement multiple times.

^b^Clicks refer to number of times a user clicks on the advertisement.

^c^Click-through rate refers to the proportion of clicks or impressions.

^d^Users screened refers to users completing the screening survey.

## Discussion

### Principal Findings

In this study of MSM at risk for HIV infection, we investigated the effectiveness of promoting free home HIV self-test kits on various internet platforms. More than half of the participants ordered a self-test kit, although only a small proportion of HIV-negative individuals reported seeking PrEP services. Our results showed that dating apps were the most efficient platform to distribute HIV self-test kits to men at high risk for HIV infection. Risk behavior, attitudes toward HIV testing and treatment, perception of HIV-related stigma, and medical mistrust were not associated with ordering a self-test kit. Finally, we recorded high prevalence of alcohol and cannabis use among participants.

Overall, information search sites performed poorly in recruiting and enrolling individuals. The site advertisement metrics showed a better click-through rate than social media and a similar number of users screened, but ultimately only a small number of individuals enrolled in the study. Search engines have a broad audience as they are available to everyone with access to the internet, and they do not require an account. In comparison, dating apps had the highest click-through rate, screening numbers, and enrollment. Users of dating apps are more likely to be MSM and engage in high-risk behaviors, which could explain the higher engagement with the promoted study advertisements. Consequently, dating apps may be more cost-efficient in enrolling select individuals compared to other platforms. Using search engines for promotion may reach higher numbers of individuals, but dating apps achieved higher interaction with the promotion message in this study.

Another important difference between platforms that may have affected individual site performance is the type of advertisement message. Social media and dating apps use blast advertisements with images and text, whereas search engines use text-only promotional content. Researchers attempting to identify the best type of advertisement to reach MSM through the internet for free at-home HIV testing [29] showed that the click-through rate for a text-only advertisement on Google was 0.38%, whereas that for advertisements with images, such as the ones used in social media and dating apps, was higher, between 0.77% and 2%.

There is a lack of published data regarding the performance of promotional campaigns to enroll participants or promote HIV prevention messages. This limits our capacity to make comparisons with similar campaigns. Our data showed that the cost of enrolling individuals from dating apps is lower compared to that for social media and information search sites. This is mainly due to the higher engagement and higher number of participants enrolled through dating apps. Future studies should collect and report advertisement campaign metrics as well as the costs of enrollment per participant screened and enrolled, which can allow for a better evaluation of the cost-effectiveness of different platforms.

### Secondary Findings

Our study demonstrated that HIV self-testing can reach individuals at high risk. We enrolled Latino and Black MSM at a high risk for HIV infection in 10 areas with a high incidence of HIV infection. The study population included individuals with inconsistent and infrequent condom use, and nearly 25% (64/254) of them reported that they had never tested for HIV. We also identified individuals who reported a preliminary positive result, which demonstrates the capacity of HIV home testing to reach hard-to-reach populations, overcome obstacles, and increase testing. Our findings underline the importance of identifying the best possible promotional platform that will allow public health programs to reach an even larger number of individuals at risk.

Our findings did not identify any major differences between participants who ordered a kit compared to those who did not order a test kit. However, our data showed a small statistical difference in terms of the questionnaires on self-perceived stigma, as well as the participant perceptions about the risks of HIV infection. Public health stakeholders should continue their efforts to educate individuals about HIV and support vulnerable individuals against stigma.

Substance use was common among study participants, especially alcohol and cannabis use. Similarly, Westmoreland et al [30] also reported a high incidence of cannabis use (55.8%) and alcohol use (22%) among a sample of MSM, transgender men, and transgender women. Heavy alcohol use is associated with an increase in sexual behaviors that might put persons at risk for HIV acquisition and transmission [31]. Therefore, HIV prevention programs should include substance use screening and intervention services.

Medical mistrust has been associated with low intention of PrEP uptake [32,33] and poor medication adherence. Medical mistrust is also a barrier to HIV testing and causes disruptions in HIV care [34]. Study participants expressed a high level of mistrust toward medical providers and institutions. However, that did not seem to affect self-test kit ordering in our study. Additional research is needed to evaluate how medical mistrust may impact HIV testing and PrEP uptake.

Regarding PrEP, participants reported being informed of its benefits, comfortable taking PrEP, and not embarrassed about taking PrEP; however, they did report concerns about the adverse effects and the cost of PrEP. Similar concerns have been reported by Kota et al [35] in a cohort of MSM. Although PrEP is generally considered safe, public health messages should include more information about its low frequency of adverse effects and overall safety. Further awareness about access to low-cost PrEP might improve uptake and retention [34]. There are established state-sponsored programs that offer low-cost or free PrEP through in-person or telemedicine visits or with simple delivery via regular mail [36]. Additional efforts to promote those initiatives and programs in high-incidence areas, such as in the areas included in this study, may be necessary.

### Limitations

A few limitations should be taken into consideration when interpreting our findings. The study was conducted in 9 areas with high HIV incidence; thus, the conclusions may not be generalizable to the whole country. Low enrollment and participation in waves affected our capacity to make broader comparisons between platforms and potentially between sites. In addition, we selected the most popular apps and sites as enrollment sites, grouping them into “platforms” with similar characteristics. Our goal was to investigate differences between the platforms. Thus, our findings are specific to the sites included in the campaigns.

### Conclusions

Our data suggest that certain dating apps may be an efficient way to reach young African American and Latinx MSM at high risk of HIV infection to promote the use of home HIV self-test kits. Dating apps are frequently used by many young MSM and offer a direct way of promoting HIV prevention to the target audiences. On the other hand, information search sites, such as Google, may require additional optimization for targeted messaging to be useful for HIV prevention. Results of this study could be used to inform public health agencies and stakeholders on what platforms are best to implement prevention campaigns.

New platforms, sites, and internet-based services are becoming available every day; therefore, research is necessary to evaluate the reach of public health and prevention campaigns using these new media outlets. Identifying and engaging individuals at increased risk for HIV infection in preventive care using entirely remote methods, including internet-based recruitment and remote access to preventive resources, is increasingly important and may represent the future of community-based HIV prevention.

## Appendix

Multimedia Appendix 1 Primary outcome analysis.

Multimedia Appendix 2 Sensitivity analyses.

Multimedia Appendix 3 Secondary outcomes.

## Abbreviations

MSM: men who have sex with men
PrEP: pre-exposure prophylaxis
