# Supplementary material for: Relative Effectiveness of Social Media, Dating Apps, and Information Search Sites in Promoting HIV Self-testing: Observational Cohort Study
Source: JMIR Form Res. 2022 Sep 23;6(9):e35648. doi: 10.2196/35648 (PMC9591705; doi:10.2196/35648)
Supplement: Multimedia Appendix 1 [file formative_v6i9e35648_app1.docx]

# Appendix A. Analysis of the Primary Outcome Measure

The promotional platforms and their planned implementation are provided in Table 1. There are rows corresponding to times, and 3 columns: A, B, and C, corresponding to three types of promotional platforms (social media sites, dating apps, and informational search sites).

**Table S1: Study Data Layout: A = Social Media sites, B = Dating apps, C = Informational search sites**

|  | A | B | C |
| --- | --- | --- | --- |
| Wave 1 | Facebook | Grindr/alternative | Google |
| Wave 2 | Instagram | Jack’d | Bing |

The study assumes that for a given time period $i$, the numbers of kits ordered from sites $i1, \ldots i3$ will be given by three independent Poisson processes with rates $\lambda_{i1}, \ldots, \lambda_{i3}$. Time period $i$ lasts until ~133 kits have been ordered, which will take time $t_{i}$.

The primary analysis model will thus be a Poisson regression model using time as an offset (Hardin & Hilbe, 2018), in which:

$$\log\left( o_{ij} \right)=log \left( t_{i} \right) +\alpha+\beta_{i}+\gamma_{j}+\beta\gamma_{ij}$$

where

$o_{ij}$ is the number of kits ordered by the site in Wave $i$ (i.e. time period $i$), platform type $j$.

$t_{i}$ is the time that the Wave platforms were recruiting.

$\beta_{i}$ is the main effect of wave.

$\gamma_{j}$ is the main effect of platform type.

$\beta\gamma_{ij}$ is the interaction term.

Under this model, the rate for any site $ij$ is given by:

$$rate_{ij}=exp\left( \alpha+\beta_{i}+\gamma_{j}+\beta\gamma_{ij} \right)$$

Wave 1 recruited at a lower rate than originally planned in the protocol. These recruitment obstacles led to the following modifications to the planned statistical analysis.

1. Statistical analysis based on pooling rates across waves will not be conducted because Wave 1 and Wave 2 recruited at different rates.
2. Comparison of platform rates will be done within waves rather than comparing pooled column rates.

**Table S2: Revised Study Data Layout: Exclude Wave 3**

|  | **Social Media Sites** | **Dating Apps** | **Informational search Sites** |
| --- | --- | --- | --- |
| Wave 1* | Facebook | Grindr | Google |
| Wave 2 | Instagram | Jack’d | Bing |
| * Combining original Wave 1 data from the time when Google, Facebook and Grindr were all advertising simultaneously and the data from the second phase of Wave 1. | | | |

Adapting the modifications listed above, the SAS code below was used to conduct analysis for the primary outcome. The model estimates platform rates (6 Poisson rates) and their 95% confidence intervals.

data long;

set simul;

lt = log(t);

run;

proc genmod data = long;

class row col;

model o = row | col / dist = poisson link = log offset = lt type3;

estimate "rate(1,1)" intercept **1** row **1** **0** col **1** **0** **0** row*col **1** **0** **0 0** **0** **0**;

estimate "rate(1,2)" intercept **1** row **1** **0** col **0** **1** **0** row*col **0** **1** **0 0** **0** **0**;

estimate "rate(1,3)" intercept **1** row **1** **0** col **0** **0** **1** row*col **0** **0** **1 0** **0** **0**;

estimate "rate(2,1)" intercept **1** row **0** **1** col **1** **0** **0** row*col **0** **0** **0 1** **0** **0**;

estimate "rate(2,2)" intercept **1** row **0** **1** col **0** **1** **0** row*col **0** **0** **0 0** **1** **0**;

estimate "rate(2,3)" intercept **1** row **0** **1** col **0** **0** **1** row*col **0** **0** **0 0** **0** **1**; run;

**Secondary Analyses of the Primary Outcome Measure**

In addition to estimating the platforms rates, the following analyses were planned as per the protocol.

1. Comparison of rates within a given column. Examine whether the 3 rates in a column are the same, and, if so, what is the pooled rate estimate and its confidence interval for that column.
2. Pairwise comparison of pooled rates between columns. Examine whether pooled column rates (across waves) of two columns are the same.

Following the modifications to statistical analysis, the above analyses will not be conducted. Instead, the following pairwise comparisons of column rates will be conducted within Wave 1 and Wave 2.

1. Examine whether rates of column 1 and column 2 are equal in Wave 1 and in Wave 2.
2. Examine whether rates of column 1 and column 3 are equal in Wave 1 and in Wave 2.
3. Examine whether rates of column 2 and column 3 are equal in Wave 1 and in Wave 2.

The above analyses will be conducted by specifying SAS contrast and estimate statements in the primary outcome model. Note that, each pairwise comparison yields a single p-value hence no anticipated multiple testing issues.

The SAS code below will be used to conduct the above analyses.

data long;

set simul;

lt = log(t);

run;

proc genmod data = long;

class row col;

model o = row | col / dist = poisson link = log offset = lt type3;

contrast "u11(A) = u12(B) AND u21(A) = u22(B)"

col **1** -**1** **0** row * col **1** -**1** **0** 0 **0** 0,

col **1** -**1** **0** row * col **0** 0 0 1 -**1** **0**;

contrast "u11(A) = u13(C) AND u21(A) = u23(C)"

col **1** **0** -**1** row * col **1** 0 -**1** 0 **0** 0,

col **1** **0** -**1** row * col **0** 0 0 1 **0** -**1**;

contrast "u12(B) = u13(C) AND u22(B) = u23(C)"

col **0** **1** -**1** row * col **0** 1 -**1** 0 **0** 0,

col **0** **1** -**1** row * col **0** 0 0 0 **1** -**1**;

run;
