# Supplementary material for: Relative Effectiveness of Social Media, Dating Apps, and Information Search Sites in Promoting HIV Self-testing: Observational Cohort Study
Source: JMIR Form Res. 2022 Sep 23;6(9):e35648. doi: 10.2196/35648 (PMC9591705; doi:10.2196/35648)
Supplement: Multimedia Appendix 2 [file formative_v6i9e35648_app2.docx]

# Appendix B. Sensitivity analyses for the primary outcome

Three sensitivity analyses were conducted.

One sensitivity analysis included any self-test kits ordered at any time during the study (i.e., outside of the 60-day window for the primary analysis) and by any participants in the Validated Participant population. Validated Participant population was 271 and total self-test kit ordered was 191. The total number of test kits ordered for this population was 190.

There was no statistically significant platform difference in study Wave 1, but there was a significant difference between the platforms in Wave 2 with Jack’D (dating app) as the most effective platform (order rate of 3.316 kits/day), followed by Instagram (social media) (order rate of 0.342 kits/day), and Bing as the least effective site with zero orders of self-test kit (Table B1).

| **Table B1: Primary outcome sensitivity analysis including Validated participants (n=271). Wave 3 was not included in the analytic model** | | | | | |
| --- | --- | --- | --- | --- | --- |
| **Platform** | **Site** | **Wave**** | **Days** | **Test Kits Ordered** | **Order rate** |
| **Informational** |  |  |  |  |  |
|  | Google | 1 | 81 | 19 | 0.235 |
|  | Bing | 2 | 38 | 0 | 0.000 |
|  |  |  | **119** | **19** | 0.160 |
| **Social media** |  |  |  |  |  |
|  | Facebook | 1 | 81 | 22 | 0.272 |
|  | Instagram | 2 | 38 | 13 | 0.342 |
|  |  |  | **119** | **35** | 0.294 |
| **Dating** |  |  |  |  |  |
|  | Grindr | 1 | 70 | 10 | 0.143 |
|  | Jack'd | 2 | 38 | 126 | 3.316 |
|  |  |  | **108** | **136** | 1.259 |
| **Total** |  |  | **119** | **190** | 1.597 |
| *** Combining original Wave 1 data from the time when Google, Facebook and Grindr were all advertising simultaneously and the data from the second phase of Wave 1.* | | | | | |

A second sensitivity analysis attempted to address the fact that Wave 1 occurred in two phases due to one promotional platform (Grindr) stopping all advertising. Given that the majority of the Wave 1 recruitment time occurred in the second phase, this sensitivity analysis only analyzed participants who enrolled during this portion of Wave 1. The total test kit ordered for this time window was 168.

There was no statistically significant difference between the platforms in Wave 1, but there was a significant difference between the platforms in Wave 2 with Jack’D (dating app) as the most effective platform (3.289 kit/day), followed by Instagram (social media) (0.342 kits/day) and Bing as the least effective site with zero orders (Table B2).

**Table B2: Primary outcome sensitivity analysis excluding the second phase of promotional wave 1.**

| **Platform** | **Site** | **Wave*** | **Days** | **Test Kits Ordered** | **Order rate** |
| --- | --- | --- | --- | --- | --- |
| **Informational** |  |  |  |  |  |
|  | Google | 1 | 63 | 14 | 0.222 |
|  | Bing | 2 | 38 | 0 | 0.000 |
|  |  |  | **101** | **14** |  |
| **Social media** |  |  |  |  |  |
|  | Facebook | 1 | 63 | 7 | 0.111 |
|  | Instagram | 2 | 38 | 13 | 0.342 |
|  |  |  | **101** | **20** |  |
| **Dating** |  |  |  |  |  |
|  | Grindr | 1 | 63 | 9 | 0.143 |
|  | Jack'd | 2 | 38 | 125 | 3.289 |
|  |  |  | **101** | **134** |  |
| **Total** |  |  | **101** | **168** | 1.663 |

The final sensitivity analysis assessed the impact of the COVID-19. The COVID-19 pandemic began after recruitment for Wave 2. To assess the potential impact of the pandemic on the current study, only data from participants enrolled prior to the beginning of the pandemic (March 13, 2020 — the date President Trump declared COVID-19 a Pandemic in the U.S.) were analyzed. The total test kits ordered for this time window was 147. The results of the analyses for this period stay the same.

There were no statistically significant differences between the platforms in Wave 1 even though Grindr (dating app) had zero orders. There was a significant difference between the platforms in Wave 2 with Jack’D (dating app) as the most effective platform (3.289 kit/day), followed by Instagram (social media) (0.342 kits/day) and Bing as the least effective site with zero orders in Wave 2 (Table B3).

**Table B3: Primary outcome sensitivity analysis including participants enrolled prior March 13^th^, 2020 to assess the impact of the COVID19 pandemic.**

| **Platform** | **Site** | **Wave** | **Days** | **Test Kits Ordered** | **Test Kit Ordered** |
| --- | --- | --- | --- | --- | --- |
| **Informational** |  |  |  |  |  |
|  | Google | 1 | 7 | 3 | 0.429 |
|  | Bing | 2 | 38 | 0 | 0.000 |
|  |  |  | **45** | **3** | **0.067** |
| **Social media** |  |  |  |  |  |
|  | Facebook | 1 | 7 | 6 | 0.857 |
|  | Instagram | 2 | 38 | 13 | 0.342 |
|  |  |  | **45** | **19** | **0.422** |
| **Dating** |  |  |  |  |  |
|  | Grindr | 1 | 7 | 0 | 0.000 |
|  | Jack'd | 2 | 38 | 125 | 3.289 |
|  |  |  | **45** | **125** | **2.778** |
| **Total** |  |  | **45** | **147** | **3.267** |
