# Supplementary material for: Relative Effectiveness of Social Media, Dating Apps, and Information Search Sites in Promoting HIV Self-testing: Observational Cohort Study
Source: JMIR Form Res. 2022 Sep 23;6(9):e35648. doi: 10.2196/35648 (PMC9591705; doi:10.2196/35648)
Supplement: Multimedia Appendix 3 [file formative_v6i9e35648_app3.docx]

Appendix C. Secondary outcome analyses

**Comparisons between study participants who ordered and did not order human immunodeficiency virus (HIV) home test kit in the National Institute on Drug Abuse Clinical Trials Network Social Media PrEP Study, 2020 (n=254)**

| a. **Tobacco, alcohol, prescription medications, and other substance use** | | | | | | |
| --- | --- | --- | --- | --- | --- | --- |
|  |  | **Ordered test kit (n=177)**  **n/N** | **Percent** | **Did not order test kit (n=77)**  **n/N** | **Percent** | **P-value^1^** |
| **Alcohol** | None | 52/177 | 29.4 | 16/77 | 20.8 | .350 |
|  | Problem use^2^ | 46/177 | 26.0 | 21/77 | 27.3 |  |
|  | High Risk Substance Use | 79/177 | 44.6 | 40/77 | 51.9 |  |
|  |  |  |  |  |  |  |
| **Cannabis** | None | 89/177 | 50.3 | 32/77 | 41.6 | .096 |
|  | Problem use | 37/177 | 20.9 | 12/77 | 15.6 |  |
|  | High Risk Substance Use | 51/177 | 28.8 | 33/77 | 42.8 |  |
|  |  |  |  |  |  |  |
| **Stimulants** | None | 166/177 | 93.8 | 69/77 | 89.6 | .299 |
|  | Problem Use/High Risk Substance Use^3^ | 11/177 | 6.2 | 8/77 | 10.4 |  |
|  |  |  |  |  |  |  |
| **Opioid** | None | 173/177 | 97.7 | 75/77 | 97.4 | 1.000 |
|  | Problem Use/ High Risk Substance Use^3^ | 4/177 | 2.3 | 2/77 | 2.6 |  |
|  |  |  |  |  |  |  |
| **Sedative** | None | 163/177 | 92.1 | 71/77 | 92.2 | .722 |
|  | Problem use/High Risk Substance Use^3^ | 9/177 | 5.1 | 5/77 | 6.5 |  |
|  | Missing | 5/177 | 2.8 | 1/77 | 1.3 |  |
|  |  |  |  |  |  |  |
| **Prescribed stimulant** | None | 167/177 | 94.4 | 73/77 | 94.8 | .727 |
|  | Problem use/High Risk Substance Use^3^ | 5/177 | 2.8 | 3/77 | 3.9 |  |
|  | Missing | 5/177 | 2.8 | 1/77 | 1.3 |  |

| **b. Stage of Health Behavior Change** | | | | | | |
| --- | --- | --- | --- | --- | --- | --- |
|  |  | **Ordered test kit (n=177)**  **n/N** | **Percent** | **Did not order test kit (n=77)**  **n/N** | **Percent** | **P-value^1^** |
| **Precontemplation** | *I do not see any need to regularly test for HIV* | 7/177 | 3.9 | 2/77 | 2.6 | .251 |
| **Contemplation** | *I think I should get tested for HIV regularly, but I am not sure* | 31/177 | 17.5 | 8/77 | 10.4 |  |
| **Determination** | *I am ready to start getting regularly tested for HIV* | 87/177 | 49.2 | 49/77 | 63.6 |  |
| **Action** | *I am trying to get tested regularly for HIV* | 40/177 | 22.6 | 12/77 | 15.6 |  |
| **Maintenance** | *I have been getting tested for HIV regularly over the past few years* | 12/177 | 6.8 | 6/77 | 7.8 |  |

| **c. Attitudes toward human immunodeficiency virus (HIV) testing** | | | | | | |
| --- | --- | --- | --- | --- | --- | --- |
|  |  | **Ordered test kit (n=177)**  **n/N** | **Percent** | **Did not order test kit (n=77)**  **n/N** | **Percent** | **P-value^1^** |
| **Getting tested for HIV helps people feel better** | Agree | 169/177 | 95.5 | 70/77 | 90.9 | .160 |
|  | Disagree | 8/177 | 4.5 | 7/77 | 9.1 |  |
| **Getting tested for HIV helps people from getting HIV** | Agree | 148/177 | 83.6 | 62/76 | 81.6 | .717 |
|  | Disagree | 29/177 | 16.4 | 14/76 | 18.4 |  |
| **People in my life would leave if I had HIV** | Agree | 59/175 | 33.7 | 37/77 | 48.1 | **.035** |
|  | Disagree | 116/175 | 66.3 | 40/77 | 51.9 |  |
| **People who tested positive for HIV should hide it from others** | Agree | 18/177 | 10.2 | 9/77 | 11.7 | .825 |
|  | Disagree | 159/177 | 89.8 | 68/77 | 88.3 |  |
| **I would rather not know if I have HIV** | Agree | 27/177 | 15.3 | 15/77 | 19.5 | .463 |
|  | Disagree | 150/177 | 84.7 | 62/77 | 80.5 |  |

| **d. Attitudes toward human immunodeficiency virus (HIV) treatment (continuous scale from 1 [strongly disagree] to 7 [strongly agree])** | | | | | | |
| --- | --- | --- | --- | --- | --- | --- |
| **Statements** |  | **Ordered test kit (n=177)**  **Mean (SD)** | **Did not order test kit (n=77)**  **Mean (SD)** | **P-value** (***Wilcoxon rank test***) |  |  |
| I am less threatened by the idea of being HIV positive than I used to be | | 4.1 (2.0) | 3.8 (2.2) | .421 |  |  |
| I am less worried about HIV infection than I used to be | | 3.7 (2.0) | 3.5 (1.8) | .399 |  |  |
| I think HIV/AIDS is less of a problem than it used to be | | 3.9 (2.1) | 3.6 (2.1) | .413 |  |  |
| I think HIV/AIDS is a less serious threat than it used to be because of new HIV/AIDS treatments | | 4.2 (2.0) | 3.8 (2.1) | .225 |  |  |
| I am much less concerned about becoming HIV positive myself because of new HIV/AIDS treatments | | 3.4 (2.0) | 3.0 (1.9) | .274 |  |  |
| I think that condom use during sex is less necessary now that new HIV/AIDS treatments are available | | 2.4 (1.8) | 2.2 (1.5) | .971 |  |  |
| I think that someone who is HIV positive now needs to care less about condom use | | 1.9 (1.7) | 2.5 (2.1) | .064 |  |  |
| I think that the need for condom use is less than it used to be, because you can always start new treatments | | 2.5 (1.9) | 2.5 (2.0) | .767 |  |  |
| I think that someone who is HIV positive and uses new HIV/AIDS treatments can be cured | | 3.2 (1.9) | 3.6 (2.2) | .199 |  |  |
| I think that new HIV/AIDS treatments can eradicate the virus from your body | | 3.1 (1.9) | 3.8 (2.1) | **.029** |  |  |

| **e. Human immunodeficiency virus (HIV)-related stigma among study participants** | | | | | | |
| --- | --- | --- | --- | --- | --- | --- |
|  |  | **Ordered test kit (n=177)**  **n/N** | **Percent** | **Did not order test kit (n=77)**  **n/N** | **Percent** | **P-value^1^** |
| **I feel afraid of people living with HIV/AIDS** | Strongly agree | 12/177 | 6.8 | 7/77 | 9.1 | .613^‡^ |
|  | Agree | 9/177 | 5.1 | 2/77 | 2.6 |  |
|  | Somewhat agree | 21/177 | 11.9 | 9/77 | 11.7 |  |
|  | Neither agree nor disagree | 20/177 | 11.3 | 9/77 | 11.7 |  |
|  | Somewhat disagree | 11/177 | 6.2 | 6/77 | 7.8 |  |
|  | Disagree | 32/177 | 18.1 | 17/77 | 22.1 |  |
|  | Strongly disagree | 72/177 | 40.7 | 27/77 | 35.1 |  |
| **I could not be friends with someone who has HIV/AIDS** | Strongly agree | 1/177 | 0.6 | 4/77 | 5.2 | **.033^‡^** |
|  | Agree | 1/177 | 0.6 | 0 | 0 |  |
|  | Somewhat agree | 3/177 | 1.7 | 1/77 | 1.3 |  |
|  | Neither agree nor disagree | 9/177 | 5.1 | 6/77 | 7.8 |  |
|  | Somewhat disagree | 7/177 | 3.9 | 1/77 | 1.3 |  |
|  | Disagree | 26/177 | 14.7 | 19/77 | 24.7 |  |
|  | Strongly disagree | 130/177 | 73.5 | 46/77 | 59.7 |  |
| **People who get HIV/AIDS through sex or drug use got what they deserve** | Strongly agree | 2/177 | 1.1 | 0 | 0 | .332^‡^ |
|  | Agree | 2/177 | 1.1 | 1/77 | 1.3 |  |
|  | Somewhat agree | 3/177 | 1.7 | 2/77 | 2.6 |  |
|  | Neither agree nor disagree | 8/177 | 4.5 | 7/77 | 9.1 |  |
|  | Somewhat disagree | 6/177 | 3.4 | 2/77 | 2.6 |  |
|  | Disagree | 24/177 | 13.6 | 12/77 | 15.6 |  |
|  | Strongly disagree | 132/177 | 74.6 | 53/77 | 68.8 |  |
|  |  |  |  |  |  |  |
| **I feel anger toward people with HIV/AIDS** | Strongly agree | 1/177 | 0.6 | 0 | 0 | .213^‡^ |
|  | Agree | 0 | 0 | 0 | 0 |  |
|  | Somewhat agree | 0 | 0 | 0 | 0 |  |
|  | Neither agree nor disagree | 11/177 | 6.2 | 5/77 | 6.5 |  |
|  | Somewhat disagree | 3/177 | 1.7 | 0 | 0 |  |
|  | Disagree | 19/177 | 10.7 | 16/77 | 20.8 |  |
|  | Strongly disagree | 143/177 | 80.8 | 56/77 | 72.7 |  |

| **f. Medical mistrust** | | | | | | |
| --- | --- | --- | --- | --- | --- | --- |
|  |  | **Ordered test kit (n=177)**  **n/N** | **Percent** | **Did not order test kit (n=77)**  **n/N** | **Percent** | **P-value^1^** |
| ***You’d better be cautious when dealing with health care organizations*** | Strongly agree | 37/177 | 20.9 | 17/76 | 22.4 | .503^‡^ |
|  | Agree | 66/177 | 37.3 | 32/76 | 42.1 |  |
|  | Disagree | 42/177 | 23.7 | 14/76 | 18.4 |  |
|  | Strongly disagree | 32/177 | 18.1 | 13/76 | 17.1 |  |
| ***Patients have sometimes been deceived or misled by health care organizations*** | Strongly agree | 32/176 | 18.2 | 11/77 | 14.3 | .413^‡^ |
|  | Agree | 83/176 | 47.2 | 34/77 | 44.1 |  |
|  | Disagree | 33/176 | 18.7 | 22/77 | 28.6 |  |
|  | Strongly disagree | 28/176 | 15.9 | 10/77 | 13.0 |  |
| ***When health care organizations make mistakes they usually cover it up*** | Strongly agree | 29/174 | 16.7 | 10/76 | 13.2 | .222^‡^ |
|  | Agree | 83/174 | 47.7 | 29/76 | 38.1 |  |
|  | Disagree | 36/174 | 20.7 | 30/76 | 39.5 |  |
|  | Strongly disagree | 26/174 | 14.9 | 7/76 | 9.2 |  |
| ***Health care organizations have sometimes done harmful experiments on patients without their knowledge*** | Strongly agree | 30/176 | 17.0 | 14/77 | 18.2 | .413^‡^ |
|  | Agree | 73/176 | 41.5 | 25/77 | 32.5 |  |
|  | Disagree | 51/176 | 29.0 | 26/77 | 33.7 |  |
|  | Strongly disagree | 22/176 | 12.5 | 12/77 | 15.6 |  |
| ***Health care organizations don’t always keep your information totally private*** | Strongly agree | 36/175 | 20.6 | 11/76 | 14.5 | .371^‡^ |
|  | Agree | 64/175 | 36.6 | 29/76 | 38.1 |  |
|  | Disagree | 47/175 | 26.8 | 23/76 | 30.3 |  |
|  | Strongly disagree | 28/175 | 16.0 | 13/76 | 17.1 |  |
| ***Sometimes I wonder if health care organizations really know what they are doing*** | Strongly agree | 22/176 | 12.5 | 9/77 | 11.7 | .965^‡^ |
|  | Agree | 66/176 | 37.5 | 28/77 | 36.3 |  |
|  | Disagree | 60/176 | 34.1 | 30/77 | 39.0 |  |
|  | Strongly disagree | 28/176 | 15.9 | 10/77 | 13.0 |  |
| ***Mistakes are common in health care organizations*** | Strongly agree | 21/176 | 11.9 | 8/75 | 10.7 | .638^‡^ |
|  | Agree | 89/176 | 50.6 | 36/75 | 48.0 |  |
|  | Disagree | 46/176 | 26.1 | 23/75 | 30.6 |  |
|  | Strongly disagree | 20/176 | 11.4 | 8/75 | 10.7 |  |

Abbreviations:

^1^Based on Fisher’s exact test, unless otherwise noted

^‡^Based on Wilcoxon rank test

^2^Problem use (at the cutoff of 1+ points) is defined as clinically important substance use that may not be severe enough to meet criteria for a substance use disorder (High Risk Substance Use) based on the Diagnostic and Statistical Manual of Mental Disorders, 5th edition (DSM-5); High Risk Substance Use (at the cutoff of 2+ points) is defined using the standard diagnostic threshold of meeting two or more DSM criteria.

^3^Problem use and High Risk Substance Use are combined due to low numbers of participants in the High Risk Substance Use category.

^4^Stage of change is a single-item question from the Transtheoretical Model of Health Behavior Change assessing readiness to test for HIV.

**Figure S1. Opinions about Pre-exposure Prophylaxis (PrEP) among participants of the National Institute on Drug Abuse Clinical Trials Network Social Media PrEP Study, 2020. *Note: values below 5% are not labeled on the figure.***

**Figure S2. Reported barriers to Pre-exposure Prophylaxis (PrEP) uptake among participants of the National Institute on Drug Abuse Clinical Trials Network Social Media PrEP Study, 2020. *Note: values below 5% are not labeled on the figure.***

**Figure S3. Reported facilitators to Pre-exposure Prophylaxis (PrEP) uptake among participants of the National Institute on Drug Abuse Clinical Trials Network Social Media PrEP Study, 2020. *Note: values below 5% are not labeled on the figure.***
